# Evaluation of a Low Dose Ketamine in Post Tonsillectomy Pain Relief: A Randomized Trial Comparing Intravenous and Subcutaneous Ketamine in Pediatrics

**DOI:** 10.5812/aapm.4399

**Published:** 2012-09-13

**Authors:** Mihan J. Javid, Mohammad Hajijafari, Asghar Hajipour, Jalil Makarem, Zahra Khazaeipour

**Affiliations:** 1 Department of Anesthesiology, Imam Khomeini Medical Center, Tehran University of Medical Sciences (TUMS), Tehran, IR Iran; 2 Brain and Spinal Cord Injury Research Center, Imam Khomeini Medical Center, Tehran University of Medical Sciences, Tehran, IR Iran

**Keywords:** Ketamine, Pain Clinics, Pain, Postoperative, Tonsillectomy

## Abstract

**Background:**

Pain control in children is still a therapeutic dilemma. Preschool patients are affected from undesirable effects of postoperative pain more than adults. Tonsillectomy is associated with a high incidence of postoperative pain, not only complicating the recovery, but also delaying patients discharge.

**Objectives:**

Despite employing different surgical and anesthetic strategies in post-tonsillectomy pain relief, this is still a clinical problem. The study was designed to evaluate the efficacy of a low dose ketamine in post tonsillectomy pain relief.

**Patients and Methods:**

Our prospective randomized double blinded study enrolled 75 pediatric patients (3-10 years old) who were scheduled for a tonsillectomy procedure. Patients were randomly assigned to one of three groups receiving; intravenous (IV) ketamine 0.5mg/kg, subcutaneous (SC) ketamine 0.5 mg/kg and placebo at the end of the operation. Post-operative pain score was assessed using modified CHEOPS.

**Results:**

In our study we did not find any significant difference among the three groups regarding sex, age, and weight, duration of operation, hemodynamic stability, and nausea and vomiting. However, in ketamine groups, pain score and analgesic consumption were significantly lower (P < 0.00). The efficacy of the both ketamine groups was similar.

**Conclusions:**

The study demonstrated that the both subcutaneous and intravenous injections of ketamine, at the end of the operation, were safe and effective for post-tonsillectomy pain control. Ketamine reduced postoperative analgesic medications consumption without increasing the risk of complications.

## 1. Background

Tonsillectomy is associated with a high incidence of postoperative pain (1-4) not only complicating the recovery but also delaying patients discharge (5). Painful throats make the patient agitated, delay oral intake and increase hospitalization and expenses (6, 7). Pain control in children is still a therapeutic dilemma. Preschool patients are affected from undesirable effects of postoperative pain more than adults (8). It has been shown that using ketamine in children, as an analgesic could reduce the consumption of other analgesic drugs such as opioids, leading to fewer adverse effects (7, 9, 10). Both interception of nociceptive input and blockage of N-methyl-d-aspartate (NMDA) activation may be necessary for preventive analgesia. Sub anesthetic dose of ketamine, by blocking the NMDA receptors abolishes peripheral afferent noxious stimulation and also prevents central sensitization of nociceptors (11-13).

## 2. Objectives

The aim of this study was to evaluate the effect of low-dose subcutaneous (SC) and intravenous (IV) administration of ketamine, at the end of the operation, on post tonsillectomy pain relief. The preventive analgesic properties of ketamine in the first six hours of tonsillectomy were investigated. Postoperative pain score, adverse effects, hemodynamic changes, recovery from anesthesia, postoperative analgesic requirement, and complications were also evaluated.

## 3. Patients and Methods

In our prospective randomized double blinded clinical trial study with a parallel design, 75 pediatric patients, ASA class of I and II and aged 3-10, who were scheduled for a tonsillectomy procedure were enrolled. Approval for the study was obtained from the Ethics Committee of Tehran University of medical sciences. Moreover a written informed consent was obtained from the patients` family. Exclusion criteria included patients with psychiatric disorders and chronic pain syndrome. The study was conducted in Imam Khomeini Medical center Valiasr hospital from December 2010 to November 2011. A power analysis was performed to determine a sufficient sample size to establish a significant difference in the incidence of need for rescue analgesia based on the results of a preliminary study, using a p-value of 0.05 and a power of 0.8. The incidence of need for rescue analgesia was 70% in placebo group and 35% in ketamine group in our preliminary study. This determined that 25 patients per group were adequate. The patients were randomly assigned to one of the three groups of 25 patients using block randomization method. Group I received 1ml normal saline IV and S.C as well, at the end of the operation. Group II received 0.5 mg/kg ketamine as 1ml IV and 1ml normal saline S.C. Group III received 0.5 mg/kg ketamine as 1ml S.C and 1ml normal saline IV. Same technique of anesthesia was performed in all groups. Anesthesia was induced after establishment of a peripheral 24-Gauge IV catheter, ECG monitoring, pulse oximetry, NIBP and scenography. Fentanyl 2 µg/kg and atropine 0.02 mg/kg were administered as premedication. Sodium thiopental 5 mg/kg and atracurium 0.6 mg/kg were administered for induction of anesthesia. The patients were intubated using spiral cuffed oral tracheal tube. The size of OTT was determined using the formula (age/4) +3. Anesthesia was maintained with isoflurane 1% and nitrous oxide 50% in oxygen. No extra muscle relaxant was administered intra operatively. Neither opioids nor local anesthetics were administered during the operation. Ketamine and placebo was administered at the end of the operation. Ventilation was controlled mechanically. Patients received serum 0.3 NaCl with 3.3% dextrose according to 4-2-1 formulation during post-operative period. All of the operations were performed by the same surgeon and with a standardized method of surgery. All the evaluation was performed by an anesthesiologist who was blinded to the groups’ allocation. Systolic blood pressure [SBP], diastolic blood pressure [DBP], heart rate (HR) and oxygen saturation (Spo2) were recorded every five minutes. The primary outcome in this study was to evaluate postoperative pain score, while the other measured variables considered as the secondary outcome. In this double blinded trial, both the operating room anesthesiologist and the anesthesiologist who evaluated the patients postoperatively and the parents of patients were blinded to the evaluation groups. Postoperative pain score (0-10) was assessed by an anesthesiologist who was blinded to the groups allocation at 5, 15, 30, 60 and 360 minutes using standard modified CHEOPS (14) (Table 1). SBP, DBP and HR were recorded at the same times. If the pain score was more than four, meperidine 0.5 mg/kg IV was administered as a rescue analgesic. Side-effects such as hallucination, agitation, nausea, vomiting, bleeding and air way complications were recorded.

**Table 1:** Modified CHEOPS Pain Scoring

	Score (0)	Score (1)	Score (2)
Cry	No	Crying, moaning	Scream
Facial	Smile	Neutral	Grimace
Verbal	Positive Statement	Negative statement	Suffering from pain
Torso	Neutral	Variable, upright	Stretched
Legs	Neutral	Continuous Move Kicking	Stretched

### 3.1. Statistical Analysis

The analyses were performed using the SPSS, Version 11.5. P value less than 0.05 was considered significant. The distribution of quantitative data was analyzed using the one sample Kolmogorov-smirnov test, and accordingly the data among three groups was analyzed using Kruskal-Wallis test, and Pairwise Comparisons was performed using the Mann-Whitney Test. Data trend for each variable within or among groups were compared with each other by repeated measurement test, and for Pairwise Comparisons, the Least Significant Difference (LSD) Test was applied.

## 4. Results

Patients` characteristics (age, sex) were similar among groups (Table 2). Hemodynamic data and duration of operation procedure were comparable (Table 2). There was a significant difference of pain score among the groups as shown in Table 3. Pain score was much higher in the control group compared to the other two groups of S.C and IV ketamine. Pair wise Comparison of pain score between placebo group and IV ketamine or SC ketamine groups were significant (P < 0.01 at all intervals). Pairwise Comparison demonstrated that pain score in IV ketamine group was lower than SC ketamine after 5 minutes post operatively (P < 0.001) but after 1 and 6 hours post operatively, was lower in SC ketamine than IV (P value of 0.007 and 0.03 respectively). Rescue analgesic was required in 72% of patients in placebo group, 28% in IV ketamine and 32% in SC ketamine groups. Analgesic requirement (meperidine consumption) is much higher in placebo group than IV ketamine (P < 0.001) and SC ketamine (P < 0.001) while there were no significant differences in analgesic requirement between the ketamine S.C and IV groups (P = 0.9). (Figure). There was no report of hallucination, airway complications or postoperative bleeding. The Incidence of Nausea and vomiting were 12.7%, 16.3% and 15.1% for the placebo, ketamine IV and SC respectively with no significant difference (P = 0.07).

**Figure:**
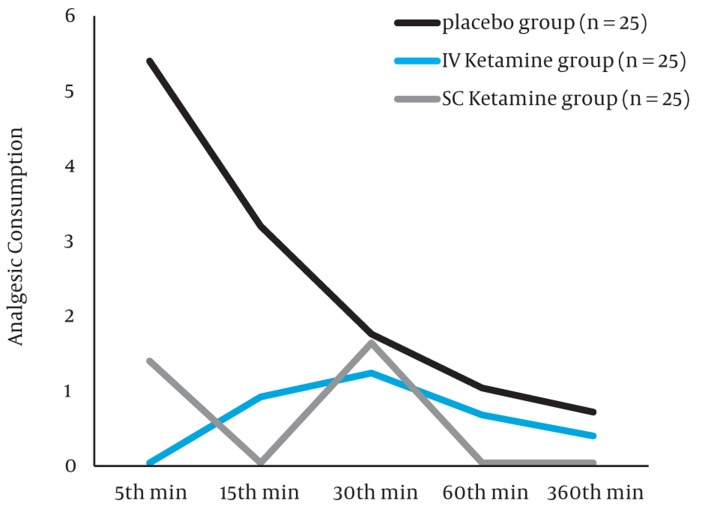
Analgesic Requirement During the Study in Three Groups

**Table 2:** Patients’ Characteristics and Duration of Operation

	Placebo Group (n = 25), Mean ± SD	IV Ketamine Group (n = 25), Mean ± SD	SC Ketamine Group (n = 25), Mean ± SD	*P* value a
Age, y	6.3 ± 1.8	6.7 ± 1.8	6.5 ± 1.8	0.637
Weight, kg	22.3 ± 6	23.3 ± 8.1	22.9 ± 6.3	0.958
Gender, Female/Male	11/14	13/12	12/13	0.8 b
HR, Beat per min	103 ± 12.5	105.6 ± 12.4	106 ± 11.1	0.807
SBP, mmHg	101 ± 9.8	103 ± 9.8	99.2 ± 11.3	0.277
DBP, mmHg	63.6 ± 8.5	64 ± 7.9	60.8 ± 8.8	0.342
Duration of operation, min	40 ± 6.4	38.8 ± 7.2	42 ± 7.8	0.234

Abbreviations: DBP, diastolic blood pressure; HR, heart rate; IV, intravenous; SBP, Systolic blood pressure; SC, subcutaneous.

^a^Kruskal Wallis Test

^b^Chi-Square Test

**Table 3:** Pain Score Among the Groups

Time After Operation	Placebo Group (n = 25), Mean ± SD	IV Ketamine Group (n = 25), Mean ± SD	SC Ketamine Group (n = 25), Mean ± SD	*P* value
5 min	5.6 ± 1.5	2.6 ± 0.8	3.8 ± 1.1	< 0.001
15 min	4.4 ± 1.2	3.3 ± 1	3.2 ± 1	< 0.001
30 min	3.9 ± 0.9	3.2 ± 1.2	3 ± 1	0.002
60 min	4 ± 0.9	3.4 ± 0.9	2.7 ± 0.7	< 0.001
360 min	3.8 ± 0.8	3.6 ± 0.8	3.2 ± 0.6	0.003

Abbreviations: IV, intravenous; SC, subcutaneous.

## 5. Discussion

Ketamine is a potent noncompetitive antagonist of NMDA at sub anesthetic dosage and it reduces the need for post-operative analgesic consumption (10). Studies in children are controversial. Some authors do not confirm the preemptive effect of ketamine on the preoperative analgesic consumption during the first hours of postoperative period in children (7, 15, 16). Our study showed that intravenous and subcutaneous administration of low dose ketamine reduced not only the post-operative pain score, but also analgesic requirement after tonsillectomy. The results of previous studies in adults using low dose intravenous ketamine preemptively, demonstrated decreased pain scores and also reduced postoperative analgesic requirement (9, 10, 17). Although, the route and the time of the drug administration were different, still the results are contradictory regarding preemptive effects (7). Some studies emphasize on preventive analgesic role of the ketamine when administered before surgical procedure, (18, 19) while some others disagree (7, 20). We demonstrated that ketamine decreased pain score and analgesic requirement after surgery when administered as a single dose at the end of the operation. Similarly, Mario Jose Da Conceic showed that using a single low dose of ketamine in pediatric patients undergoing tonsillectomy reduced opioid consumption or even eliminated the need for rescue analgesics in postoperative period regardless of administration time (7). However, this study, as so many others (7, 20) has focused on the effect of ketamine without considering the time of administration. This indicates the importance of preemptive treatment. Various routes of ketamine administration, including IV, IM, rectal, oral and subcutaneous, have been reported in different studies, among them subcutaneous administration is an accepted route (21-25). Because of its short duration of action, the analgesic effect of ketamine administered at the beginning of the operation may be terminated at the end of the procedure, allowing noxious stimulus to reach the CNS. Therefore in some investigations the bullous dose has been followed by continuous infusion (25). Likewise the same goal could be achieved by administration of ketamine at the end of the procedure. Our study showed the lower pain score in the early hours of post-operative period. This study demonstrated that SC ketamine injection(0.5 mg/kg) reduced pain score in later hours of the post-operative period (i.e. at 60 and 360 minutes after the operation) more than IV ketamine injection(0.5 mg/kg). This effect is probably because of gradual absorption of ketamine in subcutaneous administration (26). Several advantages of S.C ketamine such as lower incidence of psychomimetic side effects have been documented by keeping the plasma concentration at 150ng/ ml and even lower (22). Psychomimetic side effects have not been reported after rectal administration, which is known to result in low plasma levels (23). As shown in this study a subcutaneous injection might therefore provide analgesia without hallucinations or other psychomimetic effects (26). This study demonstrated no significant hemodynamic changes (heart rate and blood pressure) with ketamine administration. This was reported for intramuscular administration as observed in Mercadante S. and Vickers M.D investigations (27, 28). No airway problem or surgical complication occurred in our study. The beneficial effects of ketamine on the respiratory system (29) are in concordance with our findings. Ketamine may stimulate respiration by increasing the tidal volume and frequency (30). The incidence of post-operative nausea and vomiting (PONV) was a little higher in IV ketamine compared to other groups but the difference was not significant. Anyhow ketamine dose not depress laryngeal reflexes (30). Rescue analgesic requirement was higher in placebo group, than IV ketamine group, and in IV group higher than SC ketamine group. Which are similar to the findings reported by Aydin ON, et al. (6). Our main limitation in this study was to measure pain in pediatrics. Although it has been a common limitation in similar studies in children, we applied a standard method of pain measurement in children. We concluded that single dose of ketamine induces efficient analgesia with its preventive properties in pediatric post tonsillectomy patients. Also it reduced postoperative analgesic medications consumption. Both routes of administration, IV or SC at the end of the operation have similar effects and appropriate preventive analgesia, but subcutaneous route provided long term postoperative analgesia.
